# Use of WeChat applet in the management of ambulatory surgery

**DOI:** 10.1097/JS9.0000000000000304

**Published:** 2023-03-24

**Authors:** Huan Liu, Xuesheng Liu, Yao Lu

**Affiliations:** a Department of Anesthesiology; b Department of Anesthesiology, Ambulatory Surgery Center, The First Affiliated Hospital of Anhui Medical University, Hefei, China

*Dear Editor*,

Ambulatory surgery has several advantages, such as diminishing the patients’ burden, shortening the hospital stay, improving the bed turnover rate, and reducing the rate of nosocomial infection^1^,^2^. However, the accompanying issues are inevitably highlighted in the implementation of ambulatory surgery. First, the lack of effective preoperative education and preparation of patients must be seriously taken into account, as both play a pivotal role in the surgical process and contribute to a higher rate of surgery cancellations^3^. Moreover, proper management of discharged patients following ambulatory surgery increases the bed turnover rate^4^.

The WeChat applet is a revolutionary yet simple application that does not require download or installation. It improves interpersonal communication between medical surgeons and patients and makes remote management more flexible and convenient. Here, we describe the development of the WeChat applet by the Ambulatory Surgery Center of the First Affiliated Hospital of Anhui Medical University (Fig. 1).

**Figure 1:**
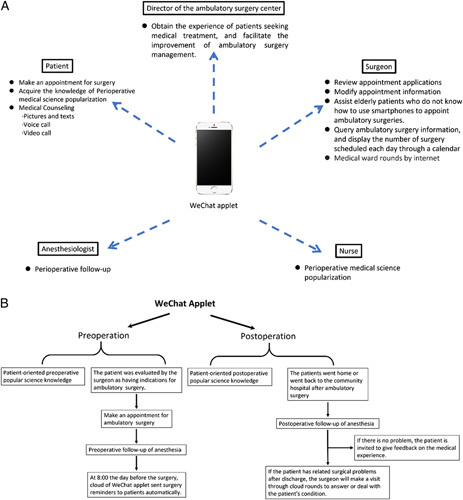
Overview of WeChat applet. (A) The users and characteristic functions of the WeChat applet. (B) The procedure of using the WeChat applet before and after surgery.

## Medical counseling

Patients undergoing ambulatory surgery can communicate with their attending physician before and after surgery by using one of three medical counseling methods provided by the applet.

In contrast to conventional medical consultation, patients can gain access to their data, communicate with their physician, and send texts or pictures in a timely manner through the applet. Voice calls can be conveniently made through the applet, especially in cases of emergency. In addition to the above methods, the applet allows video calls for consultation on personal and complex issues.

## Perioperative follow-up

By evaluating the patient’s general condition, the anesthesia regimen can be optimized to reduce the risks of adverse events and mortality. Although patients are unable to meet the anaesthesiologist in person, a preliminary assessment via video call through the applet can help the anaesthesiologist to understand the patient’s physical condition better and preliminarily estimate whether the patient meets the requirements of ambulatory surgery. In addition, the rapid discharge of patients following ambulatory surgery increases the difficulty of postoperative follow-up. Through the applet, patients can report their postoperative physical condition on the official follow-up form, which allows surgeons to understand better the patient’s postoperative recovery.

## Surgery appointment date query

The applet allows the patients who meet the operation indications after the surgeon’s evaluation to schedule their ambulatory surgery at their discretion and at any time. Additionally, patients only need to enter their hospitalization number to query the surgery date, which is convenient and fast. The applet uses the cloud server to send automatic reminder notifications to the patients reminding them of their surgery date and preoperative preparation appointment.

Another key function of the applet is to allow surgeons to access, display, and manage scheduled ambulatory operation information to optimize their workflow.

## Perioperative medical science popularization

The applet allows regular posts of popular science articles related to ambulatory surgery in order to educate patients on preoperative preparation for and postoperative management following ambulatory surgery. The aim of the function is to improve patient compliance and reduce the nursing workload in the postoperative setting. Because relevant content is easily accessible via the applet, it can be reviewed repeatedly for personal reasons, thus reducing the need for repeated consultations with the surgeon.

## Remote ward rounds

Ward rounds can be challenging because patients must be discharged within 24 h after ambulatory surgery. The applet allows surgeons to conduct ward rounds via video calls with recently discharged patients at a regular time every day, which supports better problem-solving for relevant clinical problems and ensures the safety of patients after discharge.

In summary, the WeChat-based applet helps cultivate an interactive relationship between patients, surgeons, and ambulatory surgery center managers. It is a convenient method of communication that is equipped with a variety of practical yet advanced functions that can encourage the use of Connected Health in the future. Thus, we cautiously suggest that it is an advanced and practical application that can be used to benefit more patients.

## Ethical approval

Not applicable.

## Sources of funding

None.

## Author contribution

H.L.: WeChat applet development, visualization, and writing – original draft, review, and editing; X.L.: writing – original draft preparation, reviewing, and editing; Y.L.: conceptualization, visualization, and writing – original draft preparation, reviewing, and editing.

## Conflicts of interest disclosure

The authors declare that they have no conflicts of interest.

## Research registration unique identifying number (UIN)

1. Name of the registry: not applicable.
2. Unique identifying number or registration ID: not applicable.
3. Hyperlink to your specific registration (must be publicly accessible and will be checked): not applicable.

## Guarantor

Yao Lu.

## Data availability

This communication article does not contain research data, so this item is not applicable.
